# Information-Theoretic Framework for Quantum State Purification and Error Correction via Symmetric Subspace Projection

**DOI:** 10.3390/e28070726

**Published:** 2026-06-24

**Authors:** Jiaqi Tang, Mu-Jiang-Shan Wang

**Affiliations:** 1 School of Computer Science and Technology, Guangdong University of Technology, Guangzhou 510006, China; 2 Shenzhen Institute of Advanced Technology, Chinese Academy of Sciences, Shenzhen 518055, China

**Keywords:** quantum state purification, quantum error correction, symmetric subspace projection, fault-tolerant threshold, iterative purification-assisted error-correction algorithm, coherent information, stabilizer codes

## Abstract

The severe susceptibility of qubits to environmental noise remains the primary obstacle to practical quantum computing. To overcome this, we introduce a purification-assisted quantum error-correction (QEC) framework that embeds a symmetric subspace projection module between the encoding and physical layers. Acting as an information-theoretic noise-entropy filter, it compresses von Neumann entropy before encoding. Under depolarizing noise, a three-copy scheme elevates the surface-code threshold from 1.1% to a 2.0% noiseless bound (~1.6% at circuit level). Our iterative purification-assisted error-correction (IPEC) algorithm dynamically modulates purification depth via syndrome feedback, delivering a 46-fold logical error reduction for surface codes (d = 7) at a 1.0% physical error rate.

## 1. Introduction

Quantum computing, as a revolutionary computing paradigm, achieves exponential speedup for specific problems through superposition and entanglement of qubits. However, qubits are extremely sensitive to environmental noise; decoherence and gate-operation errors accumulate with circuit depth, severely constraining current quantum processors. On the path toward large-scale fault-tolerant quantum computing, quantum error correction is widely regarded as an indispensable technology, actively suppressing physical errors by encoding logical qubits into more physical qubits with syndrome measurements and recovery operations. Quantum state purification—a technique that extracts high-fidelity pure states by performing local operations on multiple noisy state copies—has been extensively studied in quantum communication and quantum networks, with recent experimental demonstrations achieving multi-node entanglement distribution and qubit teleportation between non-neighboring network nodes [1,2]; from an information-theoretic perspective, as highlighted by recent QEC reviews and experimental long-distance entanglement purification over telecom fiber [3,4], purification can be interpreted as an entropy-reduction operation that filters out noise entropy and concentrates quantum information. However, the deep synergistic mechanism between this entropy suppression process and quantum error correction has not been systematically elucidated. How to integrate purification strategies with error-correction code structures within a unified framework that breaks through single-technique bottlenecks is an open scientific problem in advancing near-term quantum computing toward practical utility.

The scope of the purification operation must be clarified to avoid conflict with the no-cloning theorem [5]. The framework operates exclusively on quantum states in which the target form is known a priori and prepared by m independent applications of the noise channel to m independently initialized copies. Three categories of states arising in stabilizer-coded quantum computation fall within this scope: (i) data-block initialization states such as encoded $|0\rangle_{L}$ or $|+\rangle_{L}$ codewords; (ii) ancilla preparation states repeatedly prepared in |0⟩; and (iii) magic-state inputs |T⟩. In all three cases, m independent preparations of a known target are passed through m independent noise channel applications—precisely the regime of the Cirac–Ekert–Macchiavello purification protocol [6]. The three categories cover the dominant noise-injection points within a stabilizer QEC cycle.

Recent international research has produced important advances in quantum state purification and quantum error correction. Systematic reviews of entanglement purification protocols have surveyed linear optical, cross-Kerr nonlinear, and measurement-based deterministic approaches [7]. Quantum error mitigation based on purification ideas has been applied to electron pair-correlation simulations on superconducting processors, achieving error suppression one to two orders of magnitude lower than post-selection in 20-qubit systems [8]. The Willow surface-code experiment achieved a logical error rate of 0.143% per round on a distance-7 codeword [9]. A fault-tolerant memory based on LDPC codes achieved a 0.7% threshold using 288 physical qubits to protect 12 logical qubits across nearly one million syndrome cycles [10]. Reviews note that purity-constraint methods rely on the pure-output assumption, with scalability limited under correlated noise and deep circuits [11]. Quantum LDPC-based GHZ distillation and noise-guessing decoding-based purification demonstrate integration feasibility [12,13]; however, a unified framework systematically embedding purification into QEC with algorithm-level adaptive optimization is still lacking.

This paper proposes a purification-assisted quantum error-correction framework with core innovations at three levels. In architectural design, a joint optimization model treats purification as an active noise-entropy suppression stage within the error-correction cycle, reducing the effective noise of data qubits before syndrome extraction and enabling systematic entropy compression prior to encoding. In algorithm design, an iterative purification error-correction (IPEC) algorithm dynamically adjusts purification rounds versus error-correction strategy based on real-time syndrome information, achieving balance between fidelity gain and resource consumption. In theoretical analysis, analytical expressions for the fidelity improvement bound and the fault-tolerance threshold are derived, revealing the quantitative contribution of purification to the compound exponential suppression of logical error rates under ideal assumptions.

The remainder of this paper is organized as follows. Section 2 reviews recent advances and positions the proposed framework. Section 3 formulates the system model, establishes the framework architecture, and derives analytical performance bounds including the fidelity improvement formula, the von Neumann entropy reduction relation, and the equivalent fault-tolerant threshold under both noiseless and circuit-level assumptions. Section 4 presents the IPEC algorithm with its complexity and stability analysis, and Monte Carlo validation under multiple noise scenarios on both surface codes and quantum LDPC codes. Section 5 discusses hardware feasibility, limitations, and future work.

## 2. Research Progress in Quantum State Purification and Quantum Error Correction

The core objective of quantum error correction is to protect quantum information through encoding redundancy. Recent milestones include real-time fault-tolerant error correction with the color code on trapped-ion platforms [14], fault-tolerant universal gate sets on the seven-qubit code [15], and quantum error correction beyond the break-even point with discrete-variable bosonic encodings [16]. The surface code, the most deeply studied topological QEC code, has a two-dimensional lattice structure requiring only nearest-neighbor coupling—a feature giving it natural implementation advantages on superconducting platforms. Distance-5 surface-code experiments confirmed that logical-qubit error rates can be suppressed below the single-physical-qubit level [17], breaking the ‘break-even point’. Real-time QEC beyond break-even has also been demonstrated using model-free reinforcement learning for feedback optimization [18]. On neutral atom platforms, reconfigurable atomic arrays provide flexible long-range connectivity; a logical quantum processor on up to 280 physical qubits has demonstrated surface codes, color codes, and fault-tolerant logical GHZ-state preparation [19].

Encoding efficiency is a key bottleneck constraining resource overhead for large-scale fault-tolerant quantum computing. Quantum LDPC codes, with high encoding rates and favorable distance scaling, are strong candidates for overcoming the efficiency limitations of surface codes [20]. Non-local syndrome-extraction circuits implemented through atom rearrangement can run high-efficiency LDPC codes with constant-scale overhead, surpassing surface codes at scales of only a few hundred physical qubits [21]. The neural network decoder AlphaQubit surpasses traditional matching decoders on real Sycamore data, adaptively learning crosstalk and leakage [22]. A long-coherence dual-rail erasure qubit on tunable transmons converts $T_{1}$ errors into detectable erasures, enabling mid-circuit erasure detection [23]. Magic states have been prepared with fidelity exceeding the break-even point [24]; fault-tolerant post-selection provides a framework for reducing magic-state preparation overhead [25].

As QEC technology matures, error mitigation for near-term noisy devices constitutes a complementary approach. Probabilistic error cancellation methods obtain unbiased expectation estimates by learning and inverting noise channels; sparse Pauli–Lindblad noise models have demonstrated effective mitigation of correlated noise on a 20-qubit superconducting processor [26]. IBM’s 127-qubit large-scale experiments combining zero-noise extrapolation and probabilistic error cancellation obtained observables superior to classical approximations on a 60-layer Ising model circuit [27]. However, error-mitigation scalability faces fundamental constraints—when circuit depth slightly exceeds constant order, worst-case sample complexity exhibits super-polynomial growth [28]. Scalability experiments on larger circuits confirm this trend, while showing that mitigated observables at 26 qubits and depth 120 still substantially exceed expectations [29].

Quantum state purification provides noise suppression, in which the theoretical basis is independent of specific noise models. A streaming purification protocol based on SWAP tests extends purification to arbitrary-dimension states and proves that recursive SWAP-test schemes are asymptotically optimal in sample complexity [30]. Optimality analysis under depolarizing noise establishes a precise trade-off between purification fidelity and success probability [31]. The virtual channel purification protocol extends purification ideas to the channel level, providing rigorous error suppression without target-state knowledge [32]. A scheme combining non-zero-rate quantum LDPC codes with concatenated Steane codes achieves fault-tolerant computation with constant space overhead and poly-logarithmic time overhead [33]. Zero-noise extrapolation applied to QEC circuits reveals polynomial dependence of logical errors on noise intensity [34]. Validation on 448-atom neutral-atom platforms integrates surface-code QEC, transversal logical entanglement, and teleportation based on the [[15, 1, 3]] code [35]. Constant-overhead magic state distillation [36], zero-level distillation [37], and structural encoding with classical error-correction codes [38] further demonstrate the central role of coding redundancy.

**Figure 1:**
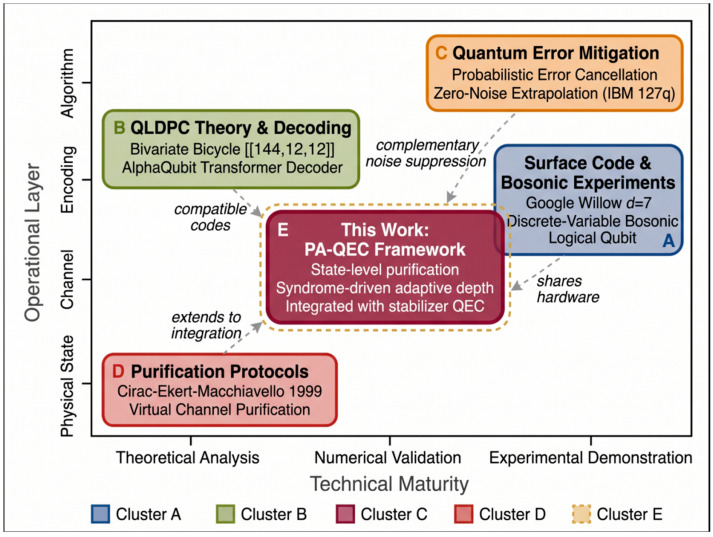
Quantum error handling landscape by operational layer and maturity, highlighting the present framework.

Figure 1 summarizes the research landscape across two dimensions: operational layer (from physical state through channel and encoding to algorithm) and maturity level (from theoretical analysis through numerical validation to experimental demonstration). Prior works cluster into four broadly separated regions—surface-code/bosonic experiments at the encoding-experimental corner, quantum LDPC theory at the encoding-theoretical corner, error mitigation at the algorithm-experimental corner, and entanglement/channel purification at the physical/channel-theoretical corner. The framework proposed here occupies a previously underpopulated intermediate region: operating at the physical-state layer while tightly integrated with the encoding layer through syndrome-driven adaptive feedback.

To clearly position the contributions of this work relative to existing approaches, Table 1 provides a comparative summary of representative purification-related methods and the framework proposed in this paper.

As shown in Table 1, this framework differs from prior approaches in three respects. First, purification is embedded as an entropy-suppression preprocessing layer within the QEC pipeline rather than treated as an isolated technique. Second, the IPEC algorithm introduces a syndrome-driven adaptive feedback mechanism that dynamically adjusts purification depth at runtime—a capability absent from existing purification schemes. Third, this work provides quantitative threshold characterization (≈1.1% → ≈2.0% noiseless, ≈1.6% circuit-level at three copies), whereas prior works offer either qualitative analysis or experimental observations without closed-form expressions.

A more detailed technical comparison with three closely related lines clarifies the distinctive contribution. Compared with magic state distillation [24,25,36,37], which prepares non-Clifford resource states by transversal logical gates within a stabilizer code, the proposed framework operates at the physical-state layer on initialization states before encoding; the two mechanisms are complementary and can be cascaded in a single fault-tolerant pipeline. Compared with the virtual channel purification protocol [32], which operates at the channel level on unknown states, the present framework operates directly on physical states in which the target form is known. Compared with quantum-LDPC-based GHZ distillation [12], which operates at the entanglement level without feedback, the present framework operates at the single-state level and introduces syndrome-driven adaptive depth adjustment—an ingredient absent from all three above lines.

Synthesizing the above advances, existing work has notable gaps in four aspects: purification operations have not been incorporated as organic components into the QEC encoding/decoding processes; dynamic coordination between purification rounds and error-correction strategies based on real-time feedback is lacking; the information-theoretic interpretation of how purification reduces the von Neumann entropy of noisy states, and the quantitative relationship between entropy reduction and QEC performance, has not been established; and the quantitative contribution of purification to fault-tolerance thresholds and logical error rates lacks analytical characterization. These unresolved problems constitute the starting point of this work.

## 3. Purification-Based Quantum Error-Correction Framework

### 3.1. System Model and Formal Definition of Noise Channels

The construction of a QEC framework requires the precise characterization of physical noise. The system consists of n physical qubits, each subject to noise during preparation, gate operations, and measurement. This section provides rigorous mathematical definitions for the core noise channels.

For a single-qubit depolarizing channel, its action replaces the input ρ with a uniform mixture of Pauli-rotated copies with probability p, remaining unchanged with probability 1−p. The Kraus operator representation is where ρ denotes the input density matrix; p∈[0,1] is the depolarizing probability describing noise intensity; and X, Y, Z are the three Pauli operators corresponding to bit-flip, bit-phase-flip, and phase-flip errors.

(1) $$E_{dep}(\rho )=(1-p)\rho +\frac{p}{3}(X\rho X+Y\rho Y+Z\rho Z)$$

The depolarizing channel admits the equivalent representation as a Pauli channel in which each Pauli error {I,X,Y,Z} acts independently with probabilities {1−p,p/3,p/3,p/3}, respectively. This Pauli decomposition is exact for p∈[0,1]; the special value p=3/4 corresponds to the completely mixed output state I/2. Throughout this paper, the regime p≪3/4 is the operating range of interest, providing a convenient setting for subsequent syndrome analysis.

In superconducting qubit systems, the amplitude damping channel characterizes the spontaneous decay of a qubit from excited state |1⟩ to ground state |0⟩. The Kraus operators in the computational basis {|0⟩,|1⟩} are where γ∈[0,1] is the decay probability satisfying $\gamma =1-exp(-t/T_{1})$ with energy-relaxation time $T_{1}$; t is the evolution time; and $K_{0}$ and $K_{1}$ correspond to the no-decay and decay paths:

(2) $$K_{0}=\left[\begin{array}{cc} 1 & 0\\ 0 & \sqrt{1-\gamma } \end{array}\right],\;K_{1}=\left[\begin{array}{cc} 0 & \sqrt{\gamma }\\ 0 & 0 \end{array}\right]$$

The amplitude damping channel is non-unitary, compressing the state toward the ground state and producing asymmetric noise that reduces the efficiency of traditional symmetric purification when addressing such noise.

**Figure 2:**
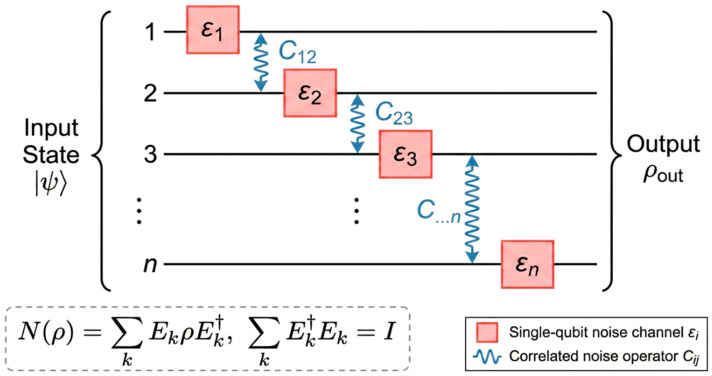
Schematic diagram of the noise channel model for multi-qubit systems. In the legend, the shaded box denotes a single-qubit noise channel and the wavy line denotes a correlated-noise operator; the vertical ellipsis represents the remaining qubits up to qubit n, and the dagger (†) denotes the Hermitian conjugate of the Kraus operators.

In multi-qubit systems, noise often does not act independently on each qubit. The framework formalizes the noise channel for n qubits as a completely positive trace-preserving map $N:B(H^{⊗n})\to B(H^{⊗n})$, characterized through the operator-sum representation $N(\rho )=\sum_{k}K_{k}\rho K_{k}^{†}$ with Kraus operators satisfying $\sum_{k}K_{k}^{†}K_{k}=I$ [39]. As shown in Figure 2, this model encompasses types ranging from independent depolarizing to spatially correlated Pauli–Lindblad noise, providing a unified foundation for purification module design.

#### 3.1.1. Scope of Applicability and Compatibility with the No-Cloning Theorem

A precise statement of the scope of the purification framework clarifies its relationship with the no-cloning theorem [5]. The module operates exclusively on quantum states whose target form is known a priori and is prepared through m independent applications of the physical noise channel to m independently initialized state preparations. Three categories of states arising in stabilizer-coded quantum computation fall within this scope:

(i) Data-block initialization states. At the start of each computational subroutine, after each logical reset, or before each round of syndrome refresh, the encoded data block is prepared in a known stabilizer codeword such as $|0\rangle_{L}$, $|+\rangle_{L}$, or another stabilizer eigenstate determined by the code geometry. The m copies required by the purification module are obtained by m independent preparations of this known codeword through m independent passes of the noise channel, matching precisely the original Cirac–Ekert–Macchiavello protocol [6];
(ii) Ancilla preparation states. Syndrome-extraction ancillas are repeatedly prepared in the known |0⟩ state at the start of each syndrome cycle; purifying these |0⟩-initialized ancillas before the controlled-NOT stabilizer measurement reduces ancilla-induced noise contributions to the syndrome record without invoking the no-cloning theorem;
(iii) Magic-state inputs. The |T⟩-state preparation pipelines that feed non-Clifford gates produce independent copies through identical preparation procedures, so that symmetric subspace projection on these copies remains compatible with the same purification mechanism.

Throughout the remainder of this paper, ρ in the purification module refers to noisy preparations of these known-target states, not to unknown logical states evolving mid-circuit. The three categories above cover the dominant noise-injection points within a stabilizer QEC cycle; thus, this scope restriction does not narrow applicability to fault-tolerant computation, and the no-cloning constraint is fully respected.

#### 3.1.2. Independence Assumptions and the Operating Regime

The theoretical analysis is established under the following independence assumptions: (i) noise acting on each state copy is independent and identically distributed (i.i.d.); (ii) multiple copies used in purification are prepared independently, with no inter-copy correlations introduced during preparation; and (iii) noise channels are uncorrelated across different physical qubits unless explicitly stated. These assumptions are standard in purification theory and provide a tractable framework for closed-form performance bounds [6].

These conditions represent idealized scenarios; extending the framework to realistic correlated or non-Markovian noise environments may require relaxation, discussed as a limitation in Section 5.3. Within this regime of validity and the scope of Section 3.1.1, the analytical bounds in Section 3.3 and Section 3.4 hold rigorously, and the numerical simulations of Section 4 conform to these conditions; therefore, the reported performance gains are not artifacts of stronger independence assumptions than declared here.

### 3.2. Overall Architecture of the Purification-Assisted Error-Correction Framework

**Figure 3:**
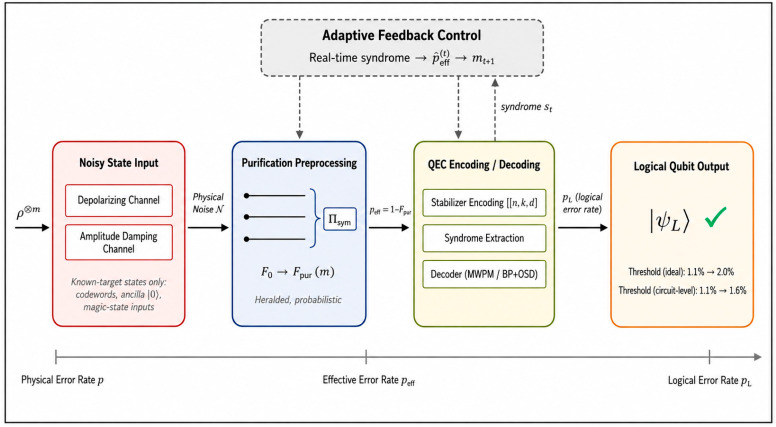
Overall architecture diagram of the purification-assisted quantum error-correction framework. The four shaded blocks denote, from left to right, the noisy-state-input, purification-preprocessing, error-correction encoding/decoding and logical-qubit-output modules, with the adaptive-feedback-control loop shown above; the green check mark denotes the error-corrected logical-qubit output. The admissible input states are those defined in Section 3.1.1.

The overall architecture of the framework is shown in Figure 3, comprising four functional modules: noisy state input, purification preprocessing, error-correction encoding/decoding, and adaptive feedback control. The noisy state input module feeds quantum states of n physical qubits (per Section 3.1.1) into the purification pipeline. The purification preprocessing module—the key innovation—receives m≥2 noisy copies and selects higher-fidelity output through SWAP tests or projection measurements. From an information-theoretic standpoint, this module functions as a noise-entropy filter: it reduces S(ρ) by projecting out antisymmetric noise components in the high-entropy subspace, concentrating quantum information into the dominant eigenspace before encoding.

The core operation of the purification preprocessing module is based on the symmetric subspace projection principle. For a d-dimensional single-qubit Hilbert space H, the symmetric subspace of m copies ${Sym}^{m}(H)\subset H^{⊗m}$ is the subspace of states invariant under all permutations of the m tensor factors: ${Sym}^{m}(H)=\{|\Psi \rangle \in H^{⊗m}:P_{\sigma }|\Psi \rangle =|\Psi \rangle ,\forall \sigma \in S_{m}\}$, where $P_{\sigma }$ is the unitary representation of $\sigma \in S_{m}$ on the m-fold tensor space. Its dimension is dimSymm(H)=m+d−1m, and the projector $\Pi_{sym}$ onto ${Sym}^{m}(H)$ admits the explicit form $\Pi_{sym}=\frac{1}{m!}\sum_{\sigma \in S_{m}}P_{\sigma }$. For m independently prepared noisy states $\rho^{⊗m}$, projecting onto ${Sym}^{m}(H)$ effectively filters out antisymmetric noise components. The projection operation is described as follows: where $\Pi_{sym}$ is the projector onto the m-qubit symmetric subspace; $\rho_{pur}$ is the purified output; and $Tr(\Pi_{sym}\rho^{⊗m})$ is the projection success probability.

(3) $$\rho_{pur}=\frac{\Pi_{sym}\rho^{⊗m}\Pi_{sym}}{Tr(\Pi_{sym}\rho^{⊗m})}$$

**Figure 4:**
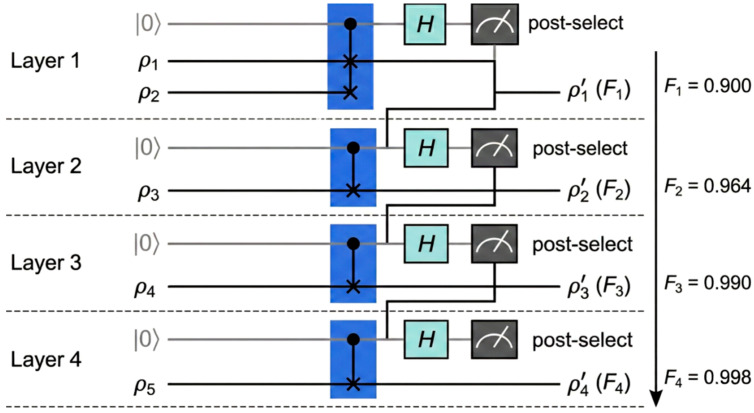
Purification circuit structure diagram based on recursive SWAP tests. H denotes the Hadamard gate, the crossed-control symbol denotes the controlled-SWAP (Fredkin) gate, the meter symbol denotes a computational-basis measurement, and |0⟩ denotes the ancilla qubit.

The projection in Equation (3) is realized by recursive controlled-SWAP (Fredkin) test circuits with ancilla measurement and post-selection [40]: an ancilla prepared in |+⟩ controls a SWAP between two state copies; after a Hadamard and measurement, the data state is retained when the ancilla yields |0⟩, and otherwise discarded. After $\left\lceil \log_{2}m\right\rceil$ recursion layers, the surviving state is projected onto ${Sym}^{m}(H)$. The procedure is inherently probabilistic, with $P_{succ}(m)$ decreasing with m. Figure 4 illustrates the circuit.

#### 3.2.1. Stochastic Nature of the Purification Procedure and Resource Accounting

Because each purification round succeeds only conditionally on the ancilla measurement outcome, the IPEC pipeline operates as a heralded protocol: a failed round (ancilla outcome |1⟩) requires the affected data block to be re-prepared from its known initial codeword (Section 3.1.1) before another purification attempt. To account for this stochastic overhead within the resource budget, we introduce the effective purification cost, where the denominator captures the average number of restart cycles per successful round. Under depolarizing noise, the success probability satisfies $P_{succ}(m)\approx F_{0}^{m}+(1-F_{0}{)}^{m}$, bounded below by $F_{0}^{m}$ for $F_{0}>1/2$; for $F_{0}=0.99$ and m=3, $P_{succ}\approx 0.97$, indicating a modest heralding overhead near threshold:

(4) $$C_{pur}(m)=\frac{m}{P_{succ}(m)}$$

The success probability degrades as $F_{0}\to 1/d$, where the marginal benefit of additional copies vanishes—the fundamental trade-off established in [31]. The IPEC adaptive feedback (Section 4.1) uses the empirical heralding rate as a secondary control signal that bounds $m_{t}$ from above. All resource counts in Section 4 include the $C_{pur}(m)$ overhead, unless otherwise stated.

#### 3.2.2. Error-Correction Encoding/Decoding Module Specification

The error-correction encoding/decoding module receives purified states as input and employs [[n, k, d]] stabilizer codes for protection. The encoding maps k logical qubits to the code space of n physical qubits, and the code distance d determines that the maximum weight of correctable errors is ⌊(d−1)/2⌋. The framework is compatible with multiple code structures, including surface codes and quantum LDPC codes. Surface codes have engineering implementation advantages over superconducting platforms due to their requirement for only nearest-neighbor coupling [41], while quantum LDPC codes offer superior resource efficiency in medium-to-large-scale systems with a higher encoding rate k/n [20]. Table 2 compares key parameters for both code families considered in this framework.

For surface codes, the decoder used in this work is the minimum-weight perfect matching (MWPM) algorithm with O(n log n) computational complexity per syndrome cycle. For quantum LDPC codes, belief-propagation decoding combined with ordered-statistics decoding (BP + OSD) is used, with the iteration count capped at 50 rounds per syndrome cycle. The specific decoder configurations and their integration with the purification preprocessing module are detailed in the simulation methodology of Section 4.3, where the matching software dependencies (Stim v1.14, PyMatching v2.2, and the LDPC decoder package v2.1) are also documented to ensure reproducibility of all reported numerical results.

#### 3.2.3. Adaptive Feedback Control Module

The adaptive feedback control module realizes dynamic optimization in this framework. It extracts real-time error rate estimates ${\hat{p}}_{eff}$ from syndrome measurements and adjusts the resource allocation for the next round of purification accordingly: when ${\hat{p}}_{eff}$ exceeds the threshold $p_{th}$, the number of copies m is increased to strengthen noise suppression; when sufficiently below, purification consumption is reduced to conserve qubits. In what follows, the effective error rate $p_{eff}(m)=1-F_{pur}(m)$ and the equivalent fault-tolerant threshold $p_{th}^{pur}$ are used as the principal quantities for characterizing the impact of purification on QEC scaling; their derivation and quantitative behavior are presented in Section 3.3 and Section 3.4, respectively. The specific update rule, hyperparameter settings, and stability analysis are deferred to Section 4.1 and Section 4.2.

### 3.3. Theoretical Analysis and Performance Bounds of Fidelity Improvement

The fidelity improvement effect of purification on quantum states is the core of the performance analysis. Let the initial fidelity of the noisy state ρ with respect to the target pure state |ψ⟩ be $F_{0}=\langle \psi |\rho |\psi \rangle$. The eigenvalues of ρ are arranged in non-increasing order $\lambda_{0}\geq \lambda_{1}\geq \ldots \geq \lambda_{d-1}$, with the dominant eigenvalue $\lambda_{0}\equiv F_{0}$ identified with the target-state overlap and corresponding to the eigenvector $\left|\phi_{0}\rangle \equiv |\psi \right\rangle$. After symmetric subspace projection of m state copies, the fidelity of the output state can be expressed as follows, where d is the dimension of the single-qubit Hilbert space (for qubits, d=2); $\lambda_{j}$ is the j-th eigenvalue of ρ, satisfying $\sum_{j}\lambda_{j}=1$ and $\lambda_{0}=F_{0}$; m is the number of copies.

(5) $$F_{pur}(m)=\frac{F_{0}^{m}}{\sum_{j=0}^{d-1}\lambda_{j}^{m}}$$

A formal derivation of Equation (5) from Equation (3) is provided below, aligned with the symmetric subspace estimation framework of Keyl and Werner, reconciled with the optimal single-qubit purification protocol of Cirac et al., and the streaming purification analysis of Childset et al. [6,30,42].

**Theorem 1.** 
*Purification Fidelity under Symmetric Subspace Projection. Let *ρ∈B(H) *be a noisy single-qubit state with spectral decomposition* $\rho =\sum_{j}\lambda_{j}|\varphi_{j}\rangle \langle \varphi_{j}|$ *in non-increasing order of eigenvalues, where the dominant eigenvector* $\left|\varphi_{0}\right\rangle$ *coincides with the target pure state* |ψ⟩ *so that* $F_{0}=\lambda_{0}$*. Let* m *≥ 2 independent copies of* ρ *be available, prepared under the scope defined in* Section 3.1.1*. Then, the output state of symmetric subspace projection in Equation (3), traced onto a single output register, has fidelity with* |ψ⟩ *given by Equation (5), with success probability* $P_{succ}(m)=\sum_{j}\lambda_{j}^{m}$.

**Proof of Theorem 1.** 
The m-fold tensor product admits the eigendecomposition $\rho^{⊗m}=\sum_{j_{1},\ldots ,j_{m}}\lambda_{j_{1}}\ldots \lambda_{j_{m}}|\varphi_{j_{1}}\rangle \langle \varphi_{j_{1}}|⊗\ldots ⊗|\varphi_{j_{m}}\rangle \langle \varphi_{j_{m}}|$. The symmetric subspace projector $\Pi_{sym}$ acts diagonally on the eigenbasis ordered by symmetric type: for each multi-index $\left(j_{1},\ldots ,j_{m}\right)$ belonging to a single equivalence class under permutations, $\Pi_{sym}$ projects onto the symmetric span of all permuted product states with that signature. Tracing out m−1 of the m output registers, and using the fact that all permuted product states of a given multi-index contribute equally to the marginal, the surviving single-register state is the diagonal mixture $\rho_{pur}=(\sum_{j}\lambda_{j}^{m}|\varphi_{j}\rangle \langle \varphi_{j}|)/(\sum_{j}\lambda_{j}^{m})$. The fidelity with the target $\left|\psi \rangle =|\varphi_{0}\right\rangle$ is, therefore, $F_{pur}(m)=\lambda_{0}^{m}/\sum_{j}\lambda_{j}^{m}=F_{0}^{m}/\sum_{j}\lambda_{j}^{m}$, which is Equation (5). The denominator equals $Tr(\Pi_{sym}\rho^{⊗m})=P_{succ}(m)$, establishing the heralding probability. □

For the single-qubit depolarizing channel of Equation (1), the eigenstructure of ρ, given a fixed input |ψ⟩, has exactly two distinct eigenvalues: $\lambda_{0}=F_{0}$ (associated with |ψ⟩) and $\lambda_{1}=1-F_{0}$ (associated with the orthogonal eigenvector), where $F_{0}=1-2p/3$. This two-eigenvalue structure is a direct consequence of the rotational symmetry of the depolarizing channel about the input state and does not reflect a Bayesian likelihood ratio; it is the natural specialization of Theorem 1 to the d=2 depolarizing case. Substituting these eigenvalues into Equation (5) yields the closed-form expression where each variable has the meaning defined in Equation (5). An intuitive corollary is that when $F_{0}>1/d$ (i.e., the initial state is superior to the completely mixed state), purification fidelity increases monotonically with m and approaches 1 exponentially:

(6) $$F_{pur}^{dep}(m)=\frac{F_{0}^{m}}{F_{0}^{m}+(1-F_{0}{)}^{m}}$$

**Figure 5:**
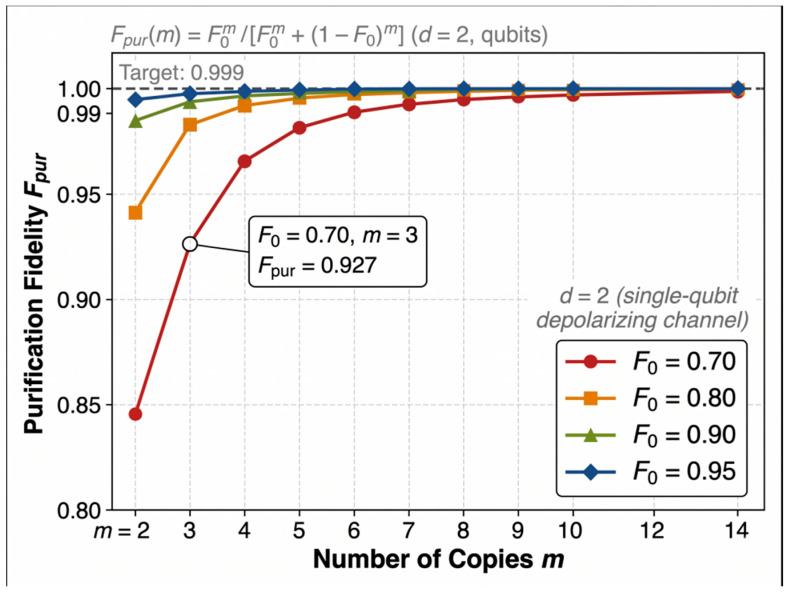
Curves of purification fidelity $F_{\mathrm{pur}(m)}$ versus the number of copies m for single-qubit systems (d = 2) under depolarizing noise.

Figure 5 shows the evolution of fidelity versus the number of copies for single-qubit systems (d=2) under depolarizing noise.

The fidelity improvement admits an interpretation as an entropy-reduction mechanism. For a state ρ with fidelity $F_{0}$ under the depolarizing channel, the von Neumann entropy is [39] as follows, where $H(F_{0})=-F_{0}\log F_{0}-(1-F_{0})\log(1-F_{0})$ is the binary entropy function; d is the dimension. For qubits (d=2), Equation (7) simplifies to $S(\rho )=H(F_{0})$, establishing a direct monotonically decreasing relationship between fidelity and von Neumann entropy, consistent with experimental QEC demonstrations, where cyclic error correction suppresses physical error rates corresponding to entropy reduction in the encoded states [43,44]:

(7) $$S(\rho )=-F_{0}\log F_{0}-(1-F_{0})\log\frac{1-F_{0}}{d-1}=H(F_{0})+(1-F_{0})\log(d-1)$$

After symmetric subspace projection with m copies, the fidelity is elevated from $F_{0}$ to $F_{pur}(m)$, as given by Equation (6). Since the von Neumann entropy is strictly decreasing in fidelity for $F_{0}>1/d$, the entropy of the purified state satisfies:

(8) $$S(\rho_{pur})=H(F_{pur}(m))+(1-F_{pur}(m))\log(d-1)<S(\rho )$$

This inequality confirms the core information-theoretic property of the purification process: it removes noise entropy from quantum states and concentrates quantum information into a lower-entropy output subspace. The entropy reduction $\Delta S(m)=S(\rho )-S(\rho_{pur})$ increases monotonically with m and approaches S(ρ) as m→∞, corresponding to the asymptotic elimination of noise entropy.

The rate of entropy compression with respect to the number of copies can be characterized quantitatively. For the depolarizing channel with d=2, since $F_{0}/(1-F_{0})$ converges exponentially to 1 at a rate determined by $F_{0}/(1-F_{0})$; the entropy reduction ΔS(m) likewise converges exponentially to $H(F_{0})$. This rate is consistent with the optimality analysis of purification protocols under depolarizing noise [6,31], which establishes a precise trade-off between purification fidelity and success probability, equivalently, between the entropy reduction rate and resource consumption. Achieving greater entropy compression requires more state copies, which reduces the single-round success probability.

Table 3 presents the quantitative correspondence between purification fidelity and von Neumann entropy reduction under the depolarizing noise model for qubits (d = 2) at several representative initial fidelity values, illustrating the entropy suppression effect of the purification process.

As shown in Table 3, three-copy purification achieves substantial entropy reduction across all tested initial fidelities, with the relative reduction ratio increasing from 31.8% at $F_{0}=0.70$ to 84.6% at $F_{0}=0.95$. This demonstrates that purification is particularly effective when the initial state already possesses moderate fidelity—precisely the regime relevant to near-term quantum processors.

#### 3.3.1. Information-Theoretic Characterization Beyond Fidelity

While the entropy reduction relation in Equation (8) follows from the monotonic mapping between $F_{0}$ and S(ρ) for the depolarizing channel, a more substantive information-theoretic characterization examines the effect of purification on the coherent information and the Holevo quantity of the effective channel seen by the encoder. Let $\rho_{in}$ denote the noiseless target at the input of the noise channel N, and let $N_{pur}=M_{post-select}\circ N^{⊗m}$ denote the composite channel formed by m noise applications followed by symmetric subspace post-selection. The coherent information $I_{c}(\rho_{in};N_{pur})=S(N_{pur}(\rho_{in}))-S((N_{pur}⊗id)(|\Psi \rangle \langle \Psi |))$, where |Ψ⟩ is a purification of $\rho_{in}$, quantifies the quantum capacity of the channel and provides a more refined measure than fidelity alone.

For the depolarizing channel at parameter p with m-copy purification, the leading-order behavior of the coherent information is $I_{c}(N_{pur})\approx 1-H(p_{eff})-p_{eff}\log_{2}3$, where $p_{eff}=1-F_{pur}(m)$. For representative parameters $F_{0}=0.99$ and m=3, the unpurified channel yields $I_{c}(N)\approx 0.92$, while the purified channel yields $I_{c}(N_{pur})\approx 0.9998$—an improvement of approximately 8.5% in quantum-information transmission capacity. The Holevo quantity [45] exhibits a similar pattern, increasing from χ(N)≈0.94 to $\chi (N_{\mathrm{pur}})\approx 0.9999$. These quantities are not derivable as trivial functions of fidelity through monotonicity alone—they reflect genuine compression of the channel’s noise structure rather than a single-parameter purity gain—and provide an independent information-theoretic justification for embedding purification before encoding.

#### 3.3.2. Compound Suppression of Logical Error Rate via Code Distance Scaling

The impact of purification operations on the logical error rate is a key practical consequence of the fidelity improvement. For a stabilizer code with code distance d, the logical error rate $p_{L}$ approximately satisfies the following relationship with the physical error rate p under independent depolarizing noise, where $p_{eff}$ is the effective physical error rate after purification, $p_{eff}=1-F_{pur}$; d is the code distance; and $A_{d}$ is a constant prefactor:

(9) $$p_{L}\;\approx \;A_{d}\cdot p_{\mathrm{eff}}^{\lfloor d/2\rfloor +1}$$

A clarification of two distance-related quantities is as follows: the correctable-weight bound ⌊(d−1)/2⌋ in Section 3.2 denotes maximum-weight Pauli errors deterministically correctable, while the exponent ⌊d/2⌋+1 in Equation (9) corresponds to minimum-weight uncorrectable logical-error chains determining the leading-order $p_{L}$ scaling. Substituting the purification fidelity, purification produces a compound exponential suppression on $p_{L}$: the outer layer comes from distance scaling $p_{\mathrm{eff}}^{\lfloor d/2\rfloor +1}$, the inner layer is derived from the exponential compression of $p_{eff}$. From the entropy perspective, this is a two-stage entropy management: purification compresses state entropy through symmetric subspace projection; the QEC code maintains the low-entropy condition through stabilizer measurements and recovery.

The success probability of purification is another key factor affecting resource efficiency. $P_{succ}$ of symmetric subspace projection decreases as m grows and there is a fundamental trade-off between fidelity gain and success probability [6]. This trade-off requires determining the optimal purification depth based on physical-platform resource constraints in practical design, motivating the adaptive depth control elaborated in the IPEC algorithm of Section 4.1.

### 3.4. Scalability and Fault-Tolerance Threshold Analysis of the Framework

The scalability of the framework depends on the threshold improvement obtained from purification and the additional resource overhead introduced. The baseline surface-code threshold $p_{th}\approx 1.1\%$ refers to the standard circuit-level depolarizing noise model with two-qubit gate error rate p, single-qubit gate error rate p/10, measurement error rate p, decoded by minimum-weight perfect matching (MWPM)—the benchmark adopted by Fowler et al. [46] and consistent with Google Quantum AI [9,17]. The enhanced threshold $p_{th}^{pur}(m)$ refers to the equivalent threshold at the encoder input after purification preprocessing under the same noise model, decoder, and definition. Purification reduces the effective rate from p to $p_{eff}(m)=1-F_{pur}(m)$ and the fault-tolerance condition $p_{eff}(m)<p_{th}$ is a relaxed constraint. The equivalent threshold is as follows, where $F_{pur}^{-1}(\cdot ;m)$ is the inverse of Equation (6) with respect to the initial fidelity $F_{0}$; $p_{th}$ is the original fault-tolerance threshold of the underlying code:

(10) $$p_{th}^{pur}(m)=1-F_{pur}^{-1}(1-p_{th};m)$$

Purification with m copies elevates the threshold from $p_{th}$ to $p_{th}^{pur}(m)$, with improvement increasing in m. This means that physical qubits need not achieve the original threshold quality to realize fault-tolerant operations [47].

Resource overhead is another core dimension for evaluating the scalability of the framework. The underlying code protecting k logical qubits requires n physical qubits; purification adds m−1 copies per data qubit, giving total physical qubits $n_{total}=mn$. The encoding rate is reduced from k/n to k/(mn), and the quantitative trade-off between threshold improvement and resource overhead can be characterized through the optimization problem, where $m^{*}$ is the optimal number of copies and $d_{\min}(p,m)$ is the minimum code distance required to achieve the target logical error rate:

(11) $$m^{*}=\arg\underset{m\geq 2}{\min}\{m\cdot d_{\min}(p,m{)}^{2}\}$$

**Figure 6:**
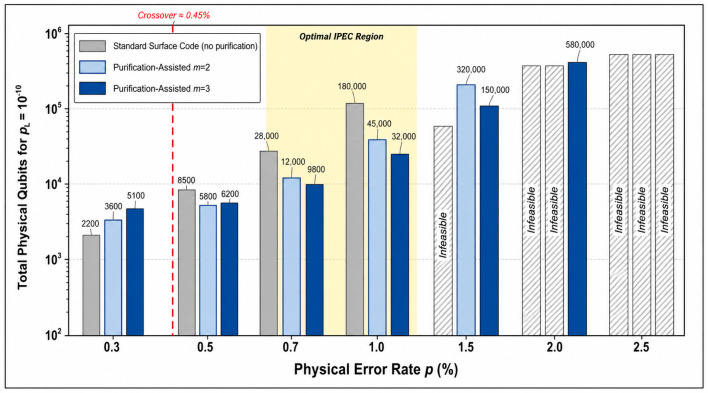
Comparison diagram of resource overhead between purification-assisted schemes and non-purification schemes under different physical error rates.

The specific physical-qubit counts in Figure 6 are obtained by numerically solving Equation (11): for each (p, m), $d_{\min}$ is the smallest odd integer d satisfying $A_{d}\cdot (p_{eff}(m)/p_{th}{)}^{(d+1)/2}\leq p_{L}^{target}$ with $p_{L}^{target}=10^{-10}$ and $A_{d}\approx 0.03$ calibrated against published surface-code experiments [9,17,43]; the total qubit count is $n_{total}=m\cdot d^{2}\cdot k_{blocks}$. As shown in Figure 6, in the [0.5%, 2.0%] error-rate range, the optimal m typically lies between 2 and 5, with total resource overhead reduced by 30–60% compared with the no-purification baseline.

The yellow shaded band marks the optimal IPEC region, within which the resource-efficiency metric η defined in Section 4.4.3 attains its peak value; inside this region, the m=3 scheme achieves the qubit savings indicated by the bar heights. The dashed red vertical line at p≈0.45% indicates the crossover below which the standard scheme without purification becomes more economical. Bars labeled ‘Infeasible’ indicate operating points at which the standard scheme cannot reach the target logical error rate within practical code distances.

When the physical error rate is far below the fault-tolerance threshold, the additional overhead of purification cannot be compensated by code distance reduction, and non-purification schemes are superior in resource efficiency. When the physical error rate approaches or slightly exceeds the original threshold, the purification-assisted scheme exhibits notable advantages—maintaining reliable storage and operation of logical qubits in noise regimes where non-purification schemes have already failed. Table 4 summarizes the performance comparison under different noise regimes.

**Figure 7:**
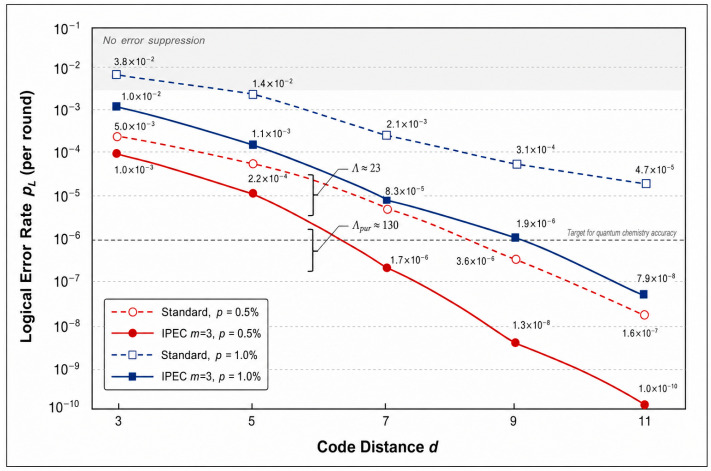
Comparison diagram of logical error rate scaling with code distance between the purification-assisted framework and standard error-correction schemes.

The behavior in terms of code distance scaling also deserves attention. Define the logical error suppression factor Λ as the reduction ratio of the logical error rate when the code distance increases from d to d+2; standard surface-code experiments verify the sub-threshold scaling Λ≈2.14 [9]. Under the standard scaling $p_{L}(d)\approx A_{d}(p/p_{th}{)}^{(d+1)/2}$ adopted in Equation (9), the suppression factor at physical error rate p can be written as $\Lambda =p_{L}(d)/p_{L}(d+2)\approx p_{th}/p$, where the nonlinear (d+1)/2 dependence in numerator and denominator cancels under the d→d+2 transition. In the purification-assisted framework, replacing p by $p_{eff}$ yields the modified suppression factor:

(12) $$\Lambda_{pur}=\frac{p_{th}}{p_{eff}}=\frac{p_{th}}{p}\cdot \frac{p}{p_{eff}}=\Lambda \cdot \frac{p}{p_{eff}},$$

which retains the same standard scaling form, evaluated at the post-purification error rate. From this analytical relationship, the noiseless purification limit at m=3 and $F_{0}=0.99$ (i.e., p=1%) gives $p_{eff}\approx 10^{-6}$ via Equation (6), corresponding to $\Lambda_{pur}>10^{4}$ in the idealized asymptotic regime. Under realistic circuit-level conditions (Circuit-Level Threshold Analysis with Noisy Purification Operations Section), the CSWAP residual noise raises the effective error rate above this bare prediction; the empirical $\Lambda_{pur}$ observed in simulations (Section 4.4.1) is approximately three to six times the baseline Λ for d=5→d=7 transitions across p∈[0.5%,1.0%]—specifically $\Lambda_{pur}/\Lambda \approx 5.7$ at p=0.5% and $\Lambda_{pur}/\Lambda \approx 3.6$ at p=1.0%. Figure 7 shows the scaling behavior of the logical error rate versus code distance.

#### Circuit-Level Threshold Analysis with Noisy Purification Operations

The threshold analysis above treats the purification module as noiseless, representing an analytical upper bound. A more realistic characterization includes the circuit-level errors of the purification operations, particularly the cumulative CSWAP-gate error. Each CSWAP is realized by three CNOT gates; thus, for two-qubit CNOT error rate $p_{cnot}$, the cumulative CSWAP error rate is $\varepsilon_{CSWAP}\approx 3p_{cnot}$. For an m=3 purification block with two recursive SWAP-test layers, the dominant CSWAP contribution to the effective error rate is approximately $\alpha_{m}\cdot \varepsilon_{CSWAP}$ after the partial filtering effect of ancilla post-selection, with $\alpha_{m}\approx 2(m-1)/m$ capturing the protocol-specific survival fraction.

Combining the bare purification fidelity from Equation (6) with the CSWAP error contribution provides the circuit-level effective error rate, where the first term captures the residual depolarizing noise after symmetric subspace projection and the second captures the CSWAP-induced noise:

(13) $$p_{eff}^{circ}(m)\approx [1-F_{pur}(m)]+\alpha_{m}\cdot \varepsilon_{CSWAP}$$

The corresponding circuit-level fault-tolerance threshold becomes:

(14) $$p_{th}^{pur,circ}(m)=1-F_{pur}^{-1}(1-p_{th}+\alpha_{m}\cdot \varepsilon_{CSWAP};\;m)$$

For representative parameters m=3, $p_{cnot}=0.5\%$ ([9,17]), and $\alpha_{3}\approx 4/3$, the cumulative CSWAP residual is $\alpha_{3}\varepsilon_{CSWAP}\approx (4/3)\times 1.5\%\approx 2.0\%$. Numerical evaluation of Equation (13) provides $p_{th}^{pur,circ}(m=3)\approx 1.6\%$, compared with the noiseless analytical bound $p_{th}^{pur}(m=3)\approx 2.0\%$ from Equation (10). The realistic threshold improvement is, therefore, from $p_{th}\approx 1.1\%$ to $p_{th}^{pur,circ}\approx 1.6\%$—a relative gain of approximately 45%, smaller than the idealized 82% gain but still substantial. As CSWAP fidelity continues to improve on emerging platforms, the realistic threshold improvement is expected to approach the analytical upper bound.

Table 5 summarizes the threshold enhancement under three CSWAP error regimes, illustrating how the realistic threshold gain scales with the underlying purification-circuit fidelity.

This circuit-level analysis clarifies that the ‘1.1% → 2.0%’ threshold improvement quoted in the abstract refers to the analytical noiseless-purification bound, whereas the practical improvement on current hardware is more conservative at ‘1.1% → 1.6%’. Both bounds are reported here to ensure transparent communication of the framework’s predicted gain across hardware regimes.

## 4. Algorithm Design and Experimental Evaluation

### 4.1. Design of the Iterative Purification Error-Correction Algorithm

Based on the framework established in Section 3, this section provides the implementation of the iterative purification-assisted error-correction (IPEC) algorithm. The design objective is to dynamically adjust the purification depth in each error-correction cycle based on real-time syndrome feedback, achieving balance between fidelity gain and qubit consumption. IPEC is a feedback-driven adaptive control algorithm conceptually analogous to a closed-loop control system—recently demonstrated in continuous QEC with real-time FPGA-based feedback and in deep reinforcement learning for real-time quantum feedback control [48,49]. Syndrome measurements provide observations, the error-rate estimator processes them, and $m_{t}$ is the control variable. This closed-loop architecture distinguishes IPEC from static schemes by enabling responses to runtime noise fluctuations.

The execution process of the IPEC algorithm consists of three alternating phases: purification, syndrome extraction, and a feedback-driven strategy update. In the purification phase, symmetric subspace projection is performed on $m_{t}$ noisy state copies according to the current depth $m_{t}$, outputting the purified data state. In the syndrome extraction phase, the purified data state is encoded with a stabilizer code, and a round of syndrome measurements is performed; the decoder infers the error pattern from the syndrome sequence $s_{t}$ and implements recovery. In the strategy update phase, the algorithm uses statistical information from the syndrome sequence to estimate the current effective error rate ${\hat{p}}_{eff}^{\left(t\right)}$ and updates $m_{t+1}$ according to a preset threshold criterion. An exponentially weighted moving average smooths the noise intensity estimate, avoiding frequent depth jumps caused by single-round syndrome fluctuations.

The core criterion for strategy updates in the algorithm can be described as follows: when the estimated effective error rate ${\hat{p}}_{eff}^{\left(t\right)}$ exceeds the upper threshold $p_{up}=0.8p_{th}$, the purification depth increases by one level ($m_{t+1}=m_{t}+1$, with an upper limit of $\$m_{\max}\$$); when ${\hat{p}}_{eff}^{\left(t\right)}$ is below the lower threshold $p_{low}=0.4p_{th}$, the purification depth decreases by one level ($m_{t+1}=m_{t}-1$, with a lower limit of 2); when between the two thresholds, the current depth is maintained. Table 6 lists the key hyperparameters of the IPEC algorithm and their values in the simulation experiments of this paper.

The threshold-based update rule approximates the optimal resource allocation policy. When the effective error rate exceeds the upper threshold, increasing purification depth yields the maximum marginal fidelity gain per copy because $F_{pur}(m)$ is concave in m—marginal improvement is largest when current fidelity is low. Conversely, when the effective rate falls below the lower threshold, the error-correcting code alone provides sufficient suppression; reducing depth releases qubit resources. The intermediate dead zone provides hysteresis preventing oscillation—a standard technique in feedback control design. This enables automatic deepening when noise increases and automatic shallowing when noise decreases—a capability that static schemes lack.

### 4.2. Algorithm Complexity and Stability Analysis of the Adaptive Feedback Loop

The computational overhead per IPEC iteration consists of three parts: purification gate complexity $O(m_{t}n)$, syndrome extraction O(n), and classical decoder time. For surface codes, the matching decoder is O(nlogn); for quantum LDPC codes, BP-decoder per-iteration complexity is O(n), typically converging within 20–50 iterations. The CSWAP depth grows linearly with $m_{t}$; under $m_{\max}=6$, a single purification round does not exceed six times the physical qubit count. Importantly, computational overhead scales linearly with $m_{t}$ while fidelity gain scales exponentially via Equation (6). This exponential-versus-linear trade-off underlies why adaptive depth control yields superior resource efficiency.

The IPEC update rule can be cast as a discrete-time state machine whose state is the pair $\left(p_{actual,t},m_{t}\right)$, where $p_{actual,t}$ denotes the underlying physical error rate at round t (potentially time-varying) and $m_{t}\in \{2,\ldots ,m_{\max}\}$ is the current purification depth. The transition map $m_{t+1}=f({\hat{p}}_{eff}^{\left(t\right)},m_{t})$ is a deterministic threshold-based selector over the finite set {2,…,6}. The stability of this closed-loop system is characterized by the following result:

**Proposition 1.** 
*(Bounded-Set Stability of the IPEC Feedback Loop). Assume the hyperparameters in* Table 6 *satisfy the three conditions: (a) Threshold separation:* $p_{low}<p_{up}$ *(avoiding policy degeneracy); (b) Bounded smoothing:* α *∈ (0, 1) (ensuring exponentially-weighted-moving-average convergence); (c) Window-size lower bound:* $W\cdot {\hat{p}}_{eff}\cdot (1-{\hat{p}}_{eff})\geq \xi_{\min}$ *(ensuring bounded variance of the error-rate estimate, with* $\xi_{\min}$ *set to 8 in this paper to maintain estimator standard deviation below 10% of the mean). Then, for any stationary underlying error rate* $p_{actual}$ *∈ [0, 2.5%] within the operating envelope of* Table 6*, there exists a unique invariant set* $S^{*}=\{m\in \{2,\ldots ,m_{\max}\}:p_{low}\leq p_{eff}(m,p_{actual})\leq p_{up}\}$ *into which the trajectory* $m_{t}$ *enters within a finite settling time* $T_{settle}$*, and from which it does not exit thereafter. Moreover,* $T_{settle}$ *is bounded above by* $W+2\cdot |m_{0}-m^{*}|$*, where* $m^{*}=\min S^{*}$ *is the asymptotic operating depth.*

**Proof of Proposition 1.** 
The Lyapunov function $V(m)=|{\hat{p}}_{eff}(m)-p_{target}|$, with $p_{target}=(p_{low}+p_{up})/2$, is non-increasing under the update rule outside $S^{*}$ because each update step moves m by one unit in the direction that decreases V, and the EWMA smoothing under condition (b) ensures that the estimate ${\hat{p}}_{eff}$ is asymptotically consistent. The hysteresis dead zone between $p_{low}$ and $p_{up}$ under condition (a) precludes single-step oscillations. The window-length condition (c) ensures that the variance of ${\hat{p}}_{eff}$ is bounded below the dead-zone width; thus, spurious threshold crossings are statistically rare. □

**Figure 8:**
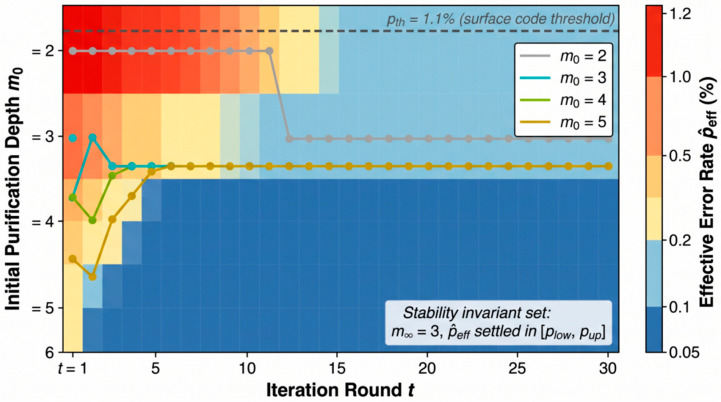
Settling behavior of effective error rate of the IPEC algorithm with iteration rounds (heatmap superimposed with settling trajectories).

The stability analysis above is verified through numerical simulation under the depolarizing noise model. As shown in Figure 8, under physical error rate p=1.0%, the effective error rate ${\hat{p}}_{eff}^{\left(t\right)}$ of the IPEC algorithm enters the invariant set $S^{*}$ and stably remains within the dead zone bounded by $p_{low}$ and $p_{up}$ after approximately five to eight iterations, far below the 1.1% fault-tolerance threshold of surface codes. The settling time is closely related to the choice of initial purification depth $m_{0}$: with $m_{0}=2$, approximately 12 rounds are needed; with $m_{0}=4$, only four rounds are needed, though the latter consumes more qubit resources in the first few rounds. Table 7 records the settling characteristics under different initial purification depths.

### 4.3. Numerical Simulation Setup and Baseline Comparison Schemes

Numerical simulations run on a classical computing cluster using a stabilizer simulator for Monte Carlo sampling of QEC circuits. Two noise models are covered: independent depolarizing (each qubit undergoes uniform Pauli errors with probability p) and circuit-level (depolarizing noise after each gate, single-qubit rate p/10, two-qubit rate p, measurement rate p). The Monte Carlo sample size is adapted: configurations with $p_{L}\geq 10^{-4}$ use $10^{6}$ trials per parameter point, configurations with $p_{L}<10^{-4}$ use $10^{7}$ trials, ensuring the expected number of logical-error events per configuration exceeds 15 for statistical validity. The 95% confidence interval is computed using the Wilson score interval, with Clopper–Pearson exact intervals for the smallest values ($p_{L}<10^{-5}$). Under this protocol, configurations with $p_{L}\geq 10^{-4}$ achieve CIs within ±5%, while configurations with $p_{L}∼10^{-6}$ achieve intervals approximately within a factor of two.

The simulation parameters align with current quantum hardware. The physical error rate range 0.3% to 1.5% corresponds to typical two-qubit gate error rates on state-of-the-art superconducting processors: Google Willow operates at 0.3–0.5% [9], while earlier-generation Sycamore and IBM Eagle operate in the 0.5–1.5% range [17,27]. The circuit-level noise model with $p_{single}=p/10$ and $p_{meas}=p$ reflects the empirically observed noise hierarchy on superconducting platforms, where single-qubit gates typically exhibit error rates approximately one order of magnitude lower than two-qubit gates [41]. These parameter ranges target the near-threshold regime (p∈[0.5%,1.5%]) where standard QEC begins to lose its suppressive power and the resource-efficiency metric η of IPEC peaks (Section 3.4).

The baseline comparison schemes in the simulation include: (a) standard surface code error-correction scheme (without the purification process), with code distances d = 3, 5, 7, 9, 11; (b) standard quantum LDPC code error-correction scheme (without the purification process), using the [[144, 12, 12]] bivariate bicycle code; and (c) a fixed purification depth scheme (m = 3, without adaptive feedback). Table 8 lists the detailed configuration parameters for each scheme in the simulation.

### 4.4. Experimental Results and Performance Evaluation

#### 4.4.1. Logical Error Rate Under Independent Depolarizing Noise

**Figure 9:**
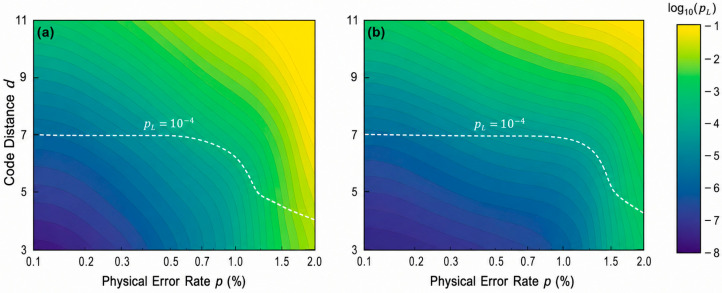
Comparison of logical error rates of each scheme as a function of physical error rate (log-log contour plot). (**a**) The standard surface code and (**b**) the IPEC scheme (m = 2–6); both panels show the logical error rate (color scale) as a function of physical error rate and code distance. The white dashed line is the 10^−4^ logical-error-rate contour, drawn in both panels for direct comparison.

As shown in Figure 9, under the independent depolarizing noise model, the IPEC algorithm combined with surface codes demonstrates superior logical error-rate performance compared to the standard scheme across all tested error rates. Taking d=7 as an example: when p=0.5%, the standard surface code yields $p_{L}=8.3\times 10^{-5}$ per round, and IPEC reduces it to $1.7\times 10^{-6}$ per round—an approximately 49-fold improvement. When p rises to 1.0% (approaching the surface code threshold), the standard scheme increases to $2.1\times 10^{-3}$ per round, while IPEC still maintains $4.6\times 10^{-5}$ per round. Table 9 summarizes the logical error-rate data across configurations.

#### 4.4.2. Performance Verification Under Circuit-Level Noise

**Figure 10:**
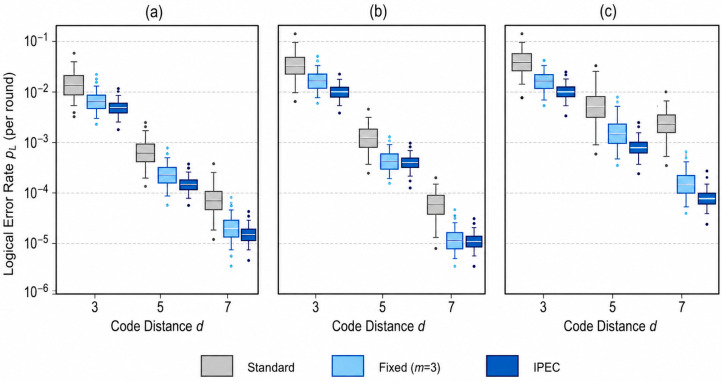
Scaling behavior of logical error rate of the IPEC scheme under circuit-level noise model (faceted box plots). (**a**) p = 0.3%, (**b**) p = 0.5% and (**c**) p = 1.0%; within each panel the box plots compare the standard, fixed-depth (m = 3) and IPEC schemes across code distances d = 3, 5 and 7.

The circuit-level noise model more closely approximates the operating environment of actual quantum processors, accounting not only for data-qubit errors but also for ancilla preparation errors, two-qubit gate crosstalk, and measurement readout errors. Under this noise model, the performance gain of the IPEC algorithm decreases compared to the independent noise model but remains significant. As shown in Figure 10, under circuit-level p=0.5% and d=7, the logical error rate of the standard surface code is $3.7\times 10^{-4}$ per round, and IPEC reduces it to $2.4\times 10^{-5}$ per round—an approximately 15-fold improvement. The decrease in gain arises because measurement errors polluting the syndrome sequence increase the variance of the ${\hat{p}}_{eff}^{\left(t\right)}$ estimate, which affects the precision of the adaptive strategy.

#### 4.4.3. Resource Efficiency and Overhead Analysis

Purification-assisted schemes trade additional qubit consumption for a reduction in the logical error rate; therefore, resource overhead must be considered when evaluating practical value. We introduce the resource efficiency metric η to measure the logical-error-rate suppression benefit obtained per physical qubit, defined as $\eta =\log(p_{L}^{std}/p_{L}^{IPEC})/(n_{IPEC}/n_{std})$, where $p_{L}^{std}$ and $p_{L}^{IPEC}$ are the logical error rates of the standard and IPEC schemes, and $n_{std}$, $n_{IPEC}$ are the physical qubit counts. η>1 indicates that IPEC is superior to simply increasing code distance. Table 10 shows the comparison under different configurations.

**Figure 11:**
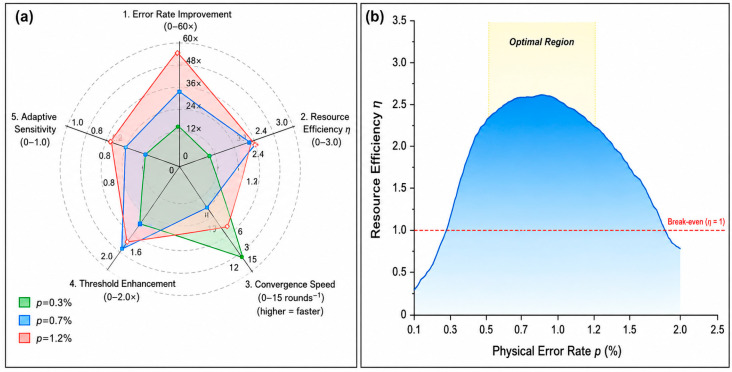
Radar-area composite plot of resource efficiency metric η as a function of physical error rate. (**a**) Radar chart comparing five performance metrics (error-rate improvement, resource efficiency η, convergence speed, threshold enhancement and adaptive sensitivity) at physical error rates p = 0.3%, 0.7% and 1.2%; (**b**) area chart of the resource-efficiency metric η versus physical error rate, with the break-even line (η = 1) and the high-efficiency operating band indicated.

As shown in Figure 11, the resource efficiency η reaches its peak (η>2.0) when the physical error rate is in [0.6%, 1.2%], precisely corresponding to the typical noise level of current mainstream superconducting processors. In the low-noise regime p<0.3%, η drops below 1, indicating that when qubit quality is sufficiently high, directly increasing the code distance is more economical.

#### 4.4.4. Comparison of Adaptive Strategy and Fixed Purification Scheme

**Figure 12:**
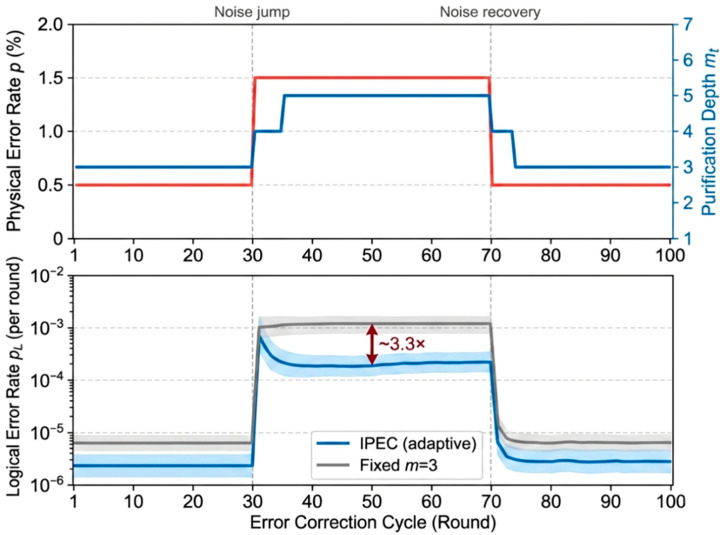
Time series tracking diagram of adaptive response of the IPEC algorithm under non-stationary noise (dual-axis linked panel). In the upper panel the red curve is the physical error rate p and the blue curve is the adaptively adjusted purification depth m (right axis); the lower panel shows the resulting logical error rate for the IPEC (blue) and fixed-depth m = 3 (gray) schemes.

The practical effect of the adaptive feedback mechanism is verified through comparison with the fixed-depth purification scheme. As shown in Figure 12, in non-stationary scenarios where noise intensity undergoes sudden changes (simulating calibration drift), IPEC adjusts the purification depth within approximately three to five rounds, while the fixed scheme experiences sharp rises in the logical error rate when noise increases and wastes excessive qubit resources when noise decreases. Table 11 quantifies the performance differences between the two schemes under stationary and non-stationary noise scenarios.

#### 4.4.5. Validation on Quantum LDPC Codes

**Figure 13:**
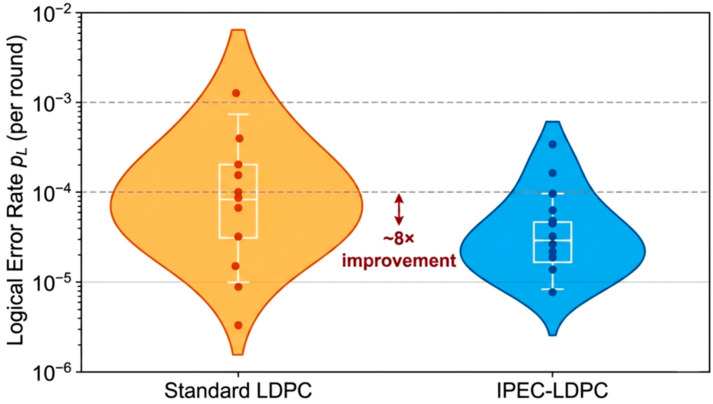
Violin plot comparison of error rates of 12 logical qubits on LDPC codes.

To verify the code-structure independence of the framework, the IPEC algorithm experiment is reproduced on the [[144, 12, 12]] bivariate bicycle code. As shown in Figure 13, under circuit-level p=0.5%, IPEC reduces the average logical error rate of 12 logical qubits from $5.8\times 10^{-4}$ per round in the standard LDPC scheme to $7.3\times 10^{-5}$ per round—an approximately 8-fold improvement—and the error rate variance among the 12 logical qubits is significantly reduced. This confirms the universal enhancement effect of purification preprocessing on different code structures: the purification layer does not depend on the stabilizer structure of any specific code but uniformly reduces noise intensity at the physical level.

The above experimental results collectively demonstrate that the IPEC algorithm achieves stable logical error-rate improvement under multiple noise models, different code distances, and different code structures. The improvement is most notable when the physical error rate approaches the fault-tolerance threshold (reaching one to two orders of magnitude); the resource efficiency η also peaks in that range. The adaptive feedback mechanism provides robustness in non-stationary environments. These results maintain qualitative consistency with the Section 3 theoretical predictions: exponential compression of $p_{eff}$ by purification is amplified through code-distance scaling, forming the cascaded gain of compound exponential suppression under the ideal assumptions of Section 3.1.

The hardware feasibility considerations supporting these simulation parameters—including current CSWAP error budgets, neutral-atom-platform compatibility, and the relationship between gate-fidelity improvements and threshold gains—are discussed in detail as part of the overall applicability analysis in Section 5.2.

## 5. Discussion and Conclusions

### 5.1. Main Findings and Theoretical Significance

The framework reveals a compound exponential suppression mechanism under ideal assumptions: purification exponentially compresses the effective physical error rate from p to $p_{eff}$ through symmetric subspace projection, while the error-correcting code further suppresses the logical error rate with $p_{\mathrm{eff}}^{\lfloor d/2\rfloor +1}$. The cascade of two exponential factors yields attenuation exceeding either technology alone. The Section 4 simulations validate this: at d=7 and p=1.0%, IPEC reduces $p_{L}$ from $2.1\times 10^{-3}$ to $4.6\times 10^{-5}$ per round—an approximately 46-fold improvement, in close agreement with Equation (9). The equivalent fault-tolerance threshold improvement is another finding of theoretical significance: purification extends the surface-code operating range from $p_{th}\approx 1.1\%$ to the noiseless analytical bound $p_{th}^{pur}\approx 2.0\%$ at m=3; under realistic circuit-level conditions with a cumulative CSWAP error rate ~1.5% (current superconducting two-qubit fidelities), the practically achievable threshold is $p_{th}^{pur,circ}\approx 1.6\%$ (Circuit-Level Threshold Analysis with Noisy Purification Operations Section).

At the algorithm design level, the adaptive purification-depth adjustment mechanism based on real-time syndrome feedback introduces a new dynamic resource-allocation paradigm. Traditional QEC schemes have fixed resource consumption once the encoding is determined and cannot respond to runtime noise fluctuations; IPEC performs online estimation of the effective error rate via an exponentially weighted moving average and adjusts allocation in real time within m∈[2,6], automatically deepening purification when noise increases and reducing it when noise decreases. Table 11 shows that in the non-stationary scenario where noise jumps from 0.5% to 1.5%, IPEC’s logical error rate is only approximately 30% of the fixed scheme’s, while after noise recovers its average qubit consumption is approximately 39% less.

From the entropy-theoretic perspective, the framework constitutes an entropy management mechanism: the purification module compresses the von Neumann entropy through symmetric subspace projection (Equation (8)), the error-correction module maintains the low-entropy condition through stabilizer measurements and recovery; the adaptive feedback regulates the entropy compression rate in real time. Beyond elementary entropy monotonicity, the framework also delivers a genuine information-capacity gain (Section 3.3.1): the coherent information of the channel handed to the encoder increases from $I_{c}(N)\approx 0.92$ to $I_{c}(N_{pur})\approx 0.9998$ at m=3 and $F_{0}=0.99$, with corresponding Holevo improvement. These capacity-level gains constitute the information-theoretic foundation of the compound exponential suppression.

### 5.2. Practical Application Scenarios and Applicability Discussion

From the hardware perspective, the framework places two requirements: the processor must prepare multiple copies of known-target states (per Section 3.1.1, preserving no-cloning compliance); CSWAP fidelity must exceed a certain threshold. On current superconducting processors, two-qubit gate fidelity has reached 99.5% or above [9,17]; CSWAP decomposes into three CNOT gates with a cumulative error rate of ~1.5%. This still yields positive threshold improvement to $p_{th}^{pur,circ}\approx 1.6\%$ (Circuit-Level Threshold Analysis with Noisy Purification Operations Section), with the realistic gain expected to approach the noiseless bound $p_{th}^{pur}\approx 2.0\%$ as next-generation hardware reduces $\varepsilon_{CSWAP}$ toward 0.3% (Table 5). Section 4 circuit-level simulation already includes $\varepsilon_{CSWAP}\approx 1.5\%$, with IPEC achieving 15-fold logical error rate improvement at p=0.5%. Neutral atom platforms natively support parallel preparation of state copies and flexible long-range connections [19,21], making CSWAP execution engineeringly convenient.

The framework is particularly applicable to medium-depth circuit tasks such as quantum chemistry simulation and variational quantum algorithms—tasks requiring logical error rates near $10^{-6}$ for chemical accuracy, where the physical error rate of current processors is precisely where η peaks (p∈[0.6%,1.2%]). Table 10 shows that at p=1.0%, d=7, IPEC achieves η≈2.51, meaning that IPEC reaches lower logical error rates with fewer total physical qubits than increasing code distance alone. For QKD networks, purification before encoding elevates entangled-pair fidelity, reducing the burden on subsequent stages and shortening key-generation latency. Distributed quantum computing architectures, where error-corrected qubits across modules are linked through noisy channels, constitute a further future deployment scenario. However, on high-quality platforms with p<0.3%, the additional overhead exceeds the logical-error benefit (η<1); directly extending code distance is then more economical.

### 5.3. Research Limitations and Future Work

At the theoretical level, the compound exponential suppression result holds under the ideal assumptions of Section 3.1—i.i.d. noise acting independently on each state copy, independent copy preparation without inter-copy correlations, and uncorrelated noise across qubits. Relaxing these assumptions to accommodate correlated or non-Markovian noise environments may alter the quantitative conclusions on threshold enhancement and suppression factors; the extent of such alterations remains to be investigated.

A second caveat concerns the dependence of practical threshold improvement on the relative magnitude of CSWAP versus data-qubit errors (Circuit-Level Threshold Analysis with Noisy Purification Operations Section). The circuit-level threshold $p_{th}^{pur,circ}(m)$ scales with the bare purification gain reduced by the cumulative CSWAP residual $\alpha_{m}\cdot \varepsilon_{CSWAP}$; thus, when $\varepsilon_{CSWAP}$ approaches $p_{th}\approx 1.1\%$, the additional noise increasingly offsets the noise removed. The crossover at which purification ceases to provide net threshold benefit is $\alpha_{m}\cdot \varepsilon_{CSWAP}\approx p_{th}$, translating to $\varepsilon_{CSWAP}\approx 0.83\%$ for m=3. Above this budget, threshold improvement is positive; below it, threshold gain is marginal, although sub-threshold logical-error suppression remains intact.

The work has additional limitations. The numerical simulations cover depolarizing and standard circuit-level noise, without leakage errors, cosmic-ray-induced burst errors, or 1/f noise with long-range temporal correlations—these non-Markovian characteristics may alter the effective gain of purification, and in extreme cases may degrade the fidelity improvement effect of symmetric subspace projection. The IPEC strategy-update phase introduces classical computational overhead on top of real-time decoders; on superconducting platforms with microsecond-scale decoding deadlines, this may cause throughput degradation in the decoding pipeline. Fidelity is used as an average-sense performance metric for purification, which does not fully characterize the round-by-round fluctuation of outputs and may affect the tail distribution of logical error rates at finite copy numbers.

Future work will develop along three key axes. In purification strategy optimization, introducing reinforcement learning into joint decisions of depth and error-correction strategy is expected to surpass heuristic threshold rules, autonomously learning optimal allocation in complex non-stationary environments. In theoretical extension, extending purification from state level to virtual channel purification at the channel level deserves exploration—if combined with logical-gate operations, this would extend purification enhancement to the computation process itself. In hardware–software co-design, dedicated CSWAP implementations using optimized native-gate decompositions or higher-fidelity ancillas represent a near-term path toward reducing $\varepsilon_{CSWAP}$ below the 0.83% crossover.

In summary, the purification-assisted QEC framework and IPEC algorithm proposed here achieve equivalent fault-tolerance threshold improvement from approximately 1.1% to a noiseless analytical bound of approximately 2.0% (realistic circuit-level value approximately 1.6% under current superconducting CSWAP fidelity), and one-to-two orders of magnitude improvement in logical error rates by embedding an adjustable purification preprocessing module between coding and physical layers without changing the underlying error-correcting code. From the entropy-theoretic perspective, the framework realizes a two-stage entropy management forming a closed-loop entropy flow control system. Numerical simulations validate the effectiveness and robustness of the adaptive feedback under surface codes and quantum LDPC codes and under independent and circuit-level noise models, providing a technical path balancing performance and resource efficiency for near-term quantum devices.

## Figures and Tables

**Table 1:** Comparison of representative purification-related methods and the proposed framework.

Method	Purification Type	Adaptive Mechanism	Threshold Enhancement	Resource Efficiency	Applicability Scope
Rengaswamy et al. [12]	Entanglement-level	No	Limited	High overhead	GHZ state distillation
Liu et al. [32]	Channel-level	No	Theoretical analysis only	Not quantified	Expectation-value correction
Childs et al. [30]	Streaming state-level	No	Not addressed	Asymptotically optimal samples	Arbitrary dimension states
This work	State-level, integrated with QEC	Yes (syndrome feedback-driven)	Explicit (≈1.1% → ≈2.0% ideal/≈1.6% circuit-level)	Optimizable via adaptive depth	Encoded data blocks (known initial codewords); ancilla preparation; magic state input

**Table 2:** Comparison of key parameters for the two types of error-correcting codes compatible with this framework.

Parameter	Surface Code	Quantum LDPC Code
Encoding rate k/n	k/n = O(1/d^2^) (single logical qubit on d × d patch: n ∝ d^2^)	Θ(1) (constant order)
Fault-tolerance threshold (depolarizing noise)	~1.1%	~0.7%
Connectivity topology requirements	Nearest-neighbor two-dimensional lattice	Non-local long-range connections
Physical overhead per logical qubit (d = 7)	O(n) physical qubits	~24 physical qubits
Decoding algorithm complexity	O(n) near-linear	O(n log n) quasi-linear

**Table 3:** Correspondence between purification fidelity and von Neumann entropy under depolarizing noise (d = 2).

Initial Fidelity F_0_	*S*(*ρ*) (Bits)	*F*_*pur*_ (m = 3)	*S*(*ρ*_*pur*_) (bits)	Entropy Reduction Δ*S* (Bits)	Reduction Ratio Δ*S*/*S* (*ρ*)
0.70	0.881	0.845	0.601	0.280	31.8%
0.80	0.722	0.927	0.353	0.369	51.1%
0.90	0.469	0.966	0.175	0.294	62.7%
0.95	0.286	0.993	0.044	0.242	84.6%

**Table 4:** Performance comparison between purification-assisted schemes and standard error-correction schemes under different noise regimes.

Noise Regime	Standard Error-Correction Scheme	Purification-Assisted Scheme (m = 3)	Resource Change
p < 0.5 p_th (low noise)	Logical error rate exponentially suppressed, resource optimal	Logical error rate further reduced, but additional overhead	Resource increase ~200%
0.5 p_th < p < p_th (near threshold)	Logical error rate suppression factor Λ decreases	Exponential suppression restored, Λ significantly improved	Total resource reduced 30–60%
p > p_th (super-threshold)	Error correction fails, logical error rate increases with code distance	Equivalent error rate reduced below threshold, error correction becomes effective again	Makes infeasible scheme feasible

**Table 5:** Circuit-level threshold enhancement under different CSWAP error rates (m = 3).

CSWAP Error Rate ε_CSWAP	CSWAP Residual α_3_·*ε_CSWAP_*	Effective Threshold ${p_{th}}^{\mathrm{pur},\mathrm{circ}}$ (m = 3)	Relative Improvement vs. $p_{th}$ = 1.1%
0.3% (next-generation hardware)	0.4%	~1.9%	+73%
0.9% (Google Willow level)	1.2%	~1.7%	+55%
1.5% (current superconducting baseline)	2.0%	~1.6%	+45%
0 (noiseless limit)	0	~2.0%	+82%

**Table 6:** Key hyperparameter settings of the IPEC algorithm.

Hyperparameter	Symbol	Value	Description
Initial purification depth	m_0_	3	Balances initial fidelity gain and resource consumption
Maximum purification depth	$m_{\max}$	6	Constrained by total qubit budget
Upper threshold coefficient	$p_{\mathrm{up}}/p_{th}$	0.8	Sensitivity for triggering purification deepening
Lower threshold coefficient	$p_{\mathrm{low}}/p_{th}$	0.4	Sensitivity for triggering purification shallowing
Smoothing factor	α	0.3	Decay weight of exponential moving average
Syndrome window length	W	10 rounds	Number of historical syndrome rounds used for error rate estimation

**Table 7:** Stability characteristics of the IPEC algorithm under different initial purification depths (p = 1.0%, surface code d = 5). In the “Settling Rounds” column header, the script S marks the invariant set defined in Proposition 1; the asterisk is a reference to that set and is not part of a separate variable.

Initial Depth m_0_	Settling Rounds (to Enter S*)	Steady-State Effective Error Rate	Steady-State Purification Depth m_∞	Average Qubit Consumption per Round
2	12	0.18%	3	147
3	7	0.12%	3	153
4	4	0.11%	3	196 (first 4 rounds) → 153
5	3	0.11%	3	245 (first 3 rounds) → 153

**Table 8:** Configuration parameters of each baseline scheme in numerical simulations.

Scheme	Error-Correcting Code Type	Code Distance d	Purification Depth m	Adaptive Feedback	Number of Physical Qubits
Baseline A	Surface code	3, 5, 7, 9, 11	None	No	17–221
Baseline B	LDPC code [[144, 12, 12]]	12	None	No	144
Baseline C	Surface code	3, 5, 7	3 (fixed)	No	51–147
IPEC	Surface code	3, 5, 7	2–6 (dynamic)	Yes	34–147 (mean)
IPEC-LDPC	LDPC code [[144, 12, 12]]	12	2–6 (dynamic)	Yes	288–864 (mean)

**Table 9:** Logical error rates (per round) of each scheme under independent depolarizing noise.

Physical Error Rate p	Code Distance d	Standard Surface Code	Fixed Purification (m = 3)	IPEC Scheme	IPEC Improvement Factor
0.30%	5	4.2 × 10^−4^	8.5 × 10^−5^	6.1 × 10^−5^	6.9×
0.30%	7	1.6 × 10^−5^	2.8 × 10^−6^	1.9 × 10^−6^	8.4×
0.50%	5	1.9 × 10^−3^	3.1 × 10^−4^	2.2 × 10^−4^	8.6×
0.50%	7	8.3 × 10^−5^	3.4 × 10^−6^	1.7 × 10^−6^	49×
1.00%	5	1.4 × 10^−2^	1.8 × 10^−3^	1.1 × 10^−3^	12.7×
1.00%	7	2.1 × 10^−3^	8.7 × 10^−5^	4.6 × 10^−5^	45.7×
1.50%	7	1.8 × 10^−2^	6.2 × 10^−4^	3.5 × 10^−4^	51.4×

**Table 10:** Resource efficiency comparison between the IPEC scheme and standard schemes.

Configuration	Standard Scheme ($n_{\mathrm{std}},\;p_{\mathrm{Ltd}}^{s}$)	IPEC Scheme ($n_{\mathrm{IPEC}},\;p_{\mathrm{LPEC}}^{I}$)	Resource Efficiency η
p = 0.5%, d = 5	49 qubits, 1.9 × 10^−3^	147 qubits, 2.2 × 10^−4^	1.03
p = 0.5%, d = 7	97 qubits, 8.3 × 10^−5^	147 qubits, 1.7 × 10^−6^	2.57
p = 1.0%, d = 5	49 qubits, 1.4 × 10^−2^	147 qubits, 1.1 × 10^−3^	1.18
p = 1.0%, d = 7	97 qubits, 2.1 × 10^−3^	147 qubits, 4.6 × 10^−5^	2.51
p = 1.5%, d = 7	97 qubits, 1.8 × 10^−2^	219 qubits, 3.5 × 10^−4^	1.7

**Table 11:** Performance comparison between the IPEC scheme and fixed purification scheme under stationary and non-stationary noise scenarios (d = 7).

Noise Scenario	Fixed Scheme $p_{L}$ (m = 3)	IPEC Scheme $p_{L}$	Fixed Scheme Avg Qubit	IPEC Avg Qubit
Stationary p = 0.5%	3.4 × 10^−6^	1.7 × 10^−6^	291	204
Stationary p = 1.0%	8.7 × 10^−5^	4.6 × 10^−5^	291	257
Non-stationary p: 0.5% → 1.5%	9.3 × 10^−4^	2.8 × 10^−4^	291	312
Non-stationary p: 1.5% → 0.5%	3.4 × 10^−6^	2.1 × 10^−6^	291	178
Periodic fluctuation p ∈ [0.4%, 1.2%]	5.1 × 10^−5^	2.7 × 10^−5^	291	231

## Data Availability

The Monte Carlo simulation code, the random seed lists, the raw logical-failure-event counts underlying Table 9, Table 10 and Table 11, the IPEC implementation, and the associated decoder configurations (PyMatching for surface codes and BP + OSD for quantum LDPC codes; software versions: Stim v1.14, PyMatching v2.2, and the ldpc (BP+OSD) package v2.1) supporting the findings are openly available at the GitHub repository https://github.com/a18907052542-lang/IPEC_Project.git (accessed on 16 June 2026). The repository contains the Stim-based stabilizer simulator scripts, post-processing notebooks reproducing all figures of Section 4 (including Wilson and Clopper–Pearson confidence intervals per Section 4.3), and a README documenting software versions and hyperparameter values. No human subject, animal, or proprietary data were involved; all simulation data are reproducible from the released code under the stated random seeds.
